# First-Principles Study on Structure and Stability of GP Zones in Al-Mg-Si(-Cu) Alloy

**DOI:** 10.3390/ma16113897

**Published:** 2023-05-23

**Authors:** Yue Su, Shaozhi He, Jiong Wang, Donglan Zhang, Qing Wu

**Affiliations:** 1 State Key Laboratory of Powder Metallurgy, Central South University, Changsha 410083, China; 213311014@csu.edu.cn (Y.S.); heshaozhi97@163.com (S.H.); 203301046@csu.edu.cn (D.Z.); 2 Information and Network Center, Central South University, Changsha 410083, China; wuqing@csu.edu.cn

**Keywords:** Al-Mg-Si(-Cu) alloy, GP zones, atomic configurations, first-principles calculations, point defects

## Abstract

Nanostructured Guinier–Preston (GP) zones are critical for the strength of Al-Mg-Si(-Cu) aluminum alloys. However, there are controversial reports about the structure and growth mechanism of GP zones. In this study, we construct several atomic configurations of GP zones according to the previous research. Then first-principles calculations based on density functional theory were used to investigate the relatively stable atomic structure and GP-zones growth mechanism. The results show that on the (100) plane, GP zones consist of {MgSi} atomic layers without Al atoms, and the size tends to grow up to 2 nm. Along the (100) growth direction, even numbers of {MgSi} atomic layers are more energetically favorable and there exist Al atomic layers to relieve the lattice strain. {MgSi}_2_Al_4_ is the most energetically favorable GP-zones configuration, and the substitution sequence of Cu atoms in {MgSi}_2_Al_4_ during the aging process is Al → Si → Mg. The growth of GP zones is accompanied by the increase in Mg and Si solute atoms and the decrease in Al atoms. Point defects, such as Cu atoms and vacancies, exhibit different occupation tendencies in GP zones: Cu atoms tend to segregate in the Al layer near the GP zones, while vacancies tend to be captured by the GP zones.

## 1. Introduction

The 6xxx series Al-Mg-Si(-Cu) alloy is widely used in the transportation and construction industries due to its good physical and chemical properties (such as excellent corrosion resistance, high specific strength, good formability, and weldability) [1,2,3]. Nanoscaled precipitation strengthening is a crucial method for enhancing the mechanical properties of Al-Mg-Si alloy [4,5,6]. The precipitation sequence of Al-Mg-Si alloy is as follows [1,7,8]: supersaturated solid solution (SSSS) → atomic clusters → Guinier–Preston (GP) zones → β″, β′, U1, U2, B′ → β, Si. Among these precipitates, the GP zones denote the nanoprecipitate phase, which forms in the initial stages of aging precipitation, possessing an ordered structure and perfect coherency with the matrix [9]. As an important precipitation phase, it plays a crucial role in improving the performance of Al-Mg-Si alloys. Studies by Ding et al. [10] and Zhu et al. [11] revealed that during the early stages of aging, the high-density GP-zones precipitation in Al-Mg-Si alloys significantly increases the alloy’s hardness. The main strengthening phases in Al-Mg-Si(-Cu) alloys come from early precipitation phases, from GP zones to β″ phases. Research on the early precipitation phases in Al-Mg-Si(-Cu) alloys is beneficial for a comprehensive understanding of the precipitation characteristics and processes of Al-Mg-Si(-Cu) alloys, which can enrich the research on the Al-Mg-Si(-Cu) system. GP zones were first discovered by Guinier [12] and Preston [13] in the Al-Cu system.

Up to now, considerable research has been conducted on the structure of Guinier–Preston (GP) zones, the early-stage precipitates in Al-Mg-Si alloy. The main structures found to date are the layer structures composed of Mg, Si, and Al atoms arranged on the (100) plane of Al, as well as the needle-like monoclinic structures similar to β″, also known as the pre-β″ phase [14,15]. The type of GP zone structure formed depends on the Mg:Si ratio. When the Mg:Si ratio is less than one, the needle-like pre-β″ phase is more likely to form, whereas when the Mg:Si ratio is greater than one, the layer structure of GP zones is more likely to form [16,17,18,19]. Matsuda et al. [16] reported that the spherical GP zones in Al-Mg-Si alloy are layer structures composed of alternating layers of Mg and Si atoms arranged in a periodic manner on the (100) plane of Al, with a Mg:Si ratio of one. Similarly, Murayama et al. [17] found that in Al-Mg-Si alloys with excess Si atoms, the GP zones also have a Mg:Si ratio of one. Liu et al. [18] proposed a similar {MgSi} layer structure for Al-Mg-Si(-Cu) alloys. Marioara et al. [19] used high-resolution transmission electron microscope (HRTEM) to discover a needle-like monoclinic structure of GP zones in Al-Mg-Si alloys with a Mg:Si ratio less than one. This structure is completely coherently aligned with the matrix. Sha et al. [20] observed an increase in Cu concentration during aging treatment, with higher Cu content in the β″ phase than in GP zones and clusters. Marceau et al. [21] suggested that the Mg-Si-Cu atomic clusters are actually Mg-Si atomic clusters. The three-dimensional atom probe (3DAP) study by Murayama et al. [22] demonstrated that the formation of GP zones is not associated with Cu enrichment.

Generally, in the GP zones, Mg and Si atoms exhibit an alternate appearance. Due to the atomic radius order of Mg > Al > Si, they can alleviate the strain caused by the difference in solute atomic radius relative to Al atoms, resulting in alternating bright and dark spots observed in HRTEM images [16,18]. The aforementioned studies confirmed the existence of the {MgSi} substructure in the GP zones. At present, there are two main problems: first, at the early stage of aging precipitation, the size of the precipitated phase is too small to determine its crystal structure and growth mechanism experimentally, and thus, computational methods can serve as an effective verification tool [23]; second, the local amplification effect in 3DAP measurements [24] makes it difficult to accurately measure the Al atomic content in the GP zones [18]. In such circumstances, several researchers have employed computational methods to conduct related studies [25,26,27]. Wolverton et al. [26] used first-principles calculations to determine that, in alloys with a Mg:Si ratio greater than one, the L1_0_ structure of Mg_1_Si_1_ is the most likely to form. Conversely, in alloys with a Mg:Si ratio of less than one, the pre-β″ AlMg_4_Si_6_ has the lowest formation enthalpy. Van Huis et al. [28] suggested that in alloys with a Mg:Si ratio of less than one, the pre-β″ Mg_2_Si_3_Al_6_ is the most stable structure. Whereas in alloys with a Mg:Si ratio greater than one, structures with {MgSi}_2_Al_10_, {MgSi}_1_Al_10_, Mg_3_Si_2_Al_5_, and Mg_2_Si_1_Al_3_, where Al atoms are inserted into the Mg and Si layers, are the most likely to form.

In the present work, first-principles calculations based on density functional theory (DFT) [29] were used to study the structure and stability of GP zones in Al-Mg-Si(-Cu) alloy. By utilizing DFT, we can obtain valuable chemical and energetic information. The structural features were obtained by experimental methods [28,30]. Specifically, we constructed a large number of atomic configurations of GP zones and analyzed their stability to determine the most stable configurations and growth mechanisms. Our study primarily focuses on the GP zone structures where the Mg:Si ratio is close to 1-2, such as Mg_2_Si_1_Al_1_, Mg_3_Si_2_Al_5_, Mg_10_Si_9_Al_19_, {MgSi}Al_2_, {MgSi}_3_Al_10_, and {MgSi}_5_Al_2_, among others. Moreover, we explored the interaction of point defects such as Cu atoms and vacancies with GP zones. The atomic-scale study of the GP zones using DFT is a promising approach to gaining insights into the mechanical properties of Al-Mg-Si(-Cu) alloy.

## 2. Materials and Methods

### 2.1. Atomic Model

**Figure 1:**
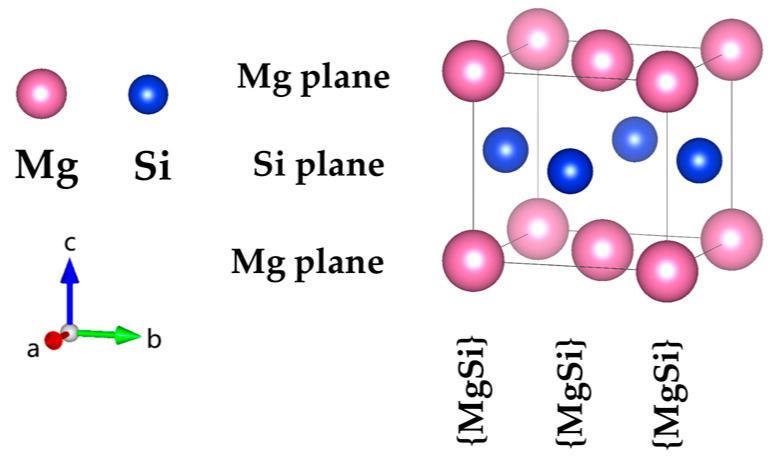
The atomic structure model of the fcc-type Mg_1_Si_1_ precipitation in the GP zones of Al-Mg-Si reported by van Huis et al. [28] and Matsuda et al. [30]. (The variables a, b, and c represent the *x*, *y*, and *z* axes, respectively, while the arrows indicate the positive directions.).

**Figure 2:**
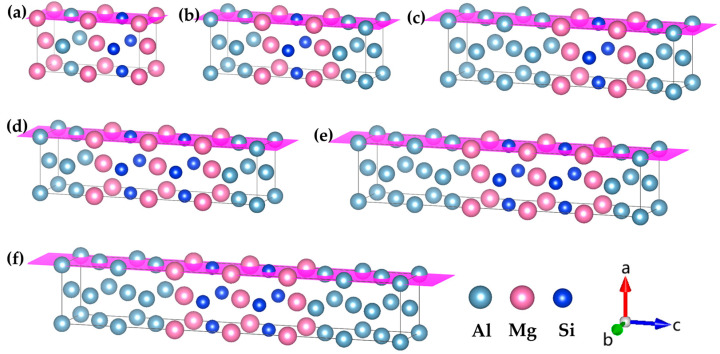
Atomic models of GP zones in Al-Mg-Si alloys with different Mg/Si/Mg cell numbers *m*. (**a**) Mg_2_Si_1_Al_1_, (**b**) Mg_2_Si_1_Al_3_ and (**c**) Mg_2_Si_1_Al_5_ are one Mg/Si/Mg unit cell with different solute atomic concentrations, (**d**) Mg_3_Si_2_Al_3_, (**e**) Mg_3_Si_2_Al_5_, and (**f**) Mg_3_Si_2_Al_7_ are two Mg/Si/Mg unit cells with different solute atomic concentrations, *m* Mg/Si/Mg unit cells and so on, the pink plane represents the {MgSi} (100) atomic plane. (The variables a, b, and c represent the *x*, *y*, and *z* axes, respectively, while the arrows indicate the positive directions.).

In this study, the atomic model of GP zones in the Al-Mg-Si alloy was constructed based on the Mg_1_Si_1_ atomic model. The fcc-type Mg_1_Si_1_ atomic-structure model of the GP-zones precipitated phase in Al-Mg-Si was reported by van Huis et al. [28] and Matsuda et al. Figure 1 shows [30]. To systematically investigate the GP zones of Al-Mg-Si alloys, we utilized a combination of cell expansion and precise adjustment of the solute atom concentration. We incrementally increased the size of the cell while carefully tuning the concentration of solute atoms to generate a series of atomic configurations with varying compositions. In addition, to investigate the presence of Al atoms in the GP zones of Al-Mg-Si alloys, (100) or (001) planes of Al atomic layers were inserted. The (100) crystal plane composed of Mg and Si atoms is known as the {MgSi} atomic layer. To model the solute atom content of {MgSi} (100) crystal plane in GP zones of Al-Mg-Si alloys, we controlled the GP-zone size and solute atom concentration. Figure 2 illustrates the atomic model of GP zones in the Al-Mg-Si alloy with different Mg/Si/Mg cell numbers, *m*. The cell composed of the Mg/Si/Mg (001) plane is represented as *m* = 1. Only the atomic model of *m* = 1, 2 are shown, and other atomic models can be deduced in this way.

**Figure 3:**
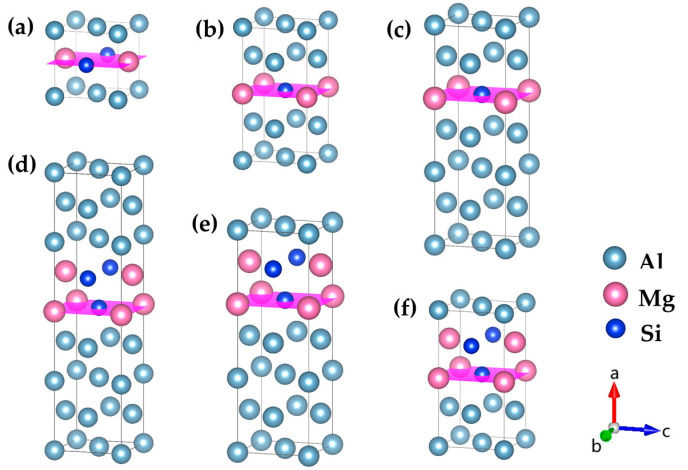
Atomic models of GP zones with different {MgSi} (100) layer numbers n in Al-Mg-Si alloys. (**a**) {MgSi}_1_Al_2_, (**b**) {MgSi}_1_Al_6_, and (**c**) {MgSi}_1_Al_10_ are monolayer {MgSi} layers with different solute atomic concentrations (*n* = 1), (**d**) {MgSi}_2_Al_12_, (**e**) {MgSi}_2_Al_8_, and (**f**) {MgSi}_2_Al_4_ are bilayer {MgSi} layers with different solute atomic concentrations (*n* = 2), *n* layers {MgSi} and so on, the pink plane represents the {MgSi} (100) atomic plane. (The variables a, b, and c represent the *x*, *y*, and *z* axes, respectively, while the arrows indicate the positive directions.)

On the basis that the solute atom concentration of {MgSi} (100) crystal plane was 100% (see Section 3.1), the number of {MgSi} mixed layers on the {MgSi} (100) direction was studied and the atomic model was constructed as shown in Figure 3. An atomic layer composed of {MgSi} (100) planes is correspondingly denoted as *n* = 1. Similarly, only the atomic models for *n* = 1, 2 are shown, and the other atomic models can be deduced in this way. We have calculated the mismatch strain values for all configurations and found that they are relatively small. Therefore, in this study, we focused primarily on analyzing the growth mechanism from an energetic perspective.

During the heat treatment of Al-Mg-Si(-Cu) alloy, a supersaturated solid solution and a certain concentration of vacancies (~0.01–0.10 at.%) will be generated [28]. In order to study the interaction of Cu atoms and vacancy site defects with the GP zones in the early stage of aging precipitation. Liu et al. [18] obtained the composition of GP zones according to the 3DAP and HRTEM results and constructed the minimum GP-zones atomic model. Therefore, in order to study this situation, we refer to the 2 × 1 × 3 GP-zones atomic model given by Liu et al., which is embedded in the Al matrix. The initial lattice parameter is 4.043 Å, the same as the Al matrix.

**Figure 4:**
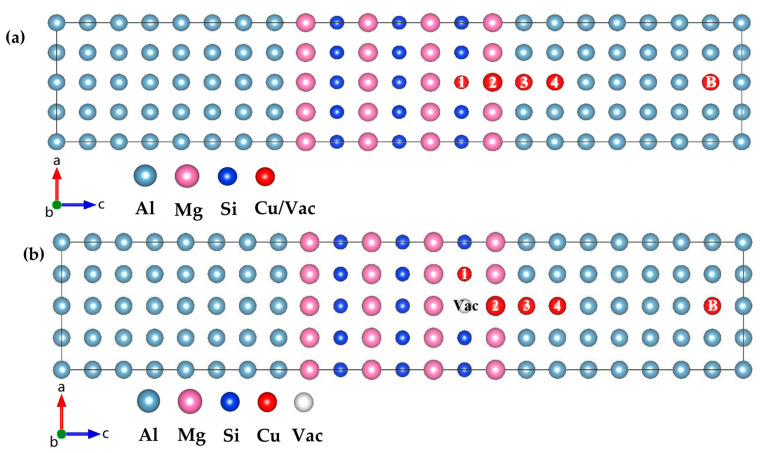
(**a**) Atomic model for calculation of point-defect occupancy propensity. (**b**) Atomic model of interaction between vacancies within GP zones and point defects. Site 1, 2, 3, 4, and B indicates that the Cu atom/Vac incorporates in the Si layer, Mg layer, Al layer nearest to the GP zones, Al layer next nearest to the GP zones, and Al matrix away from the GP zones, respectively. (The variables a, b, and c represent the *x*, *y*, and *z* axes, respectively, while the arrows indicate the positive directions.)

The occupancy tendency of point defects in materials is influenced by the energy difference between their possible locations. Point defects have different occupation tendencies in the GP zones, and vacancies strongly tend to be located within GP zones. It can be expected that some quenching vacancies may be captured by the GP zones. It is of great interest to study how these captured vacancies within the GP zones interact with Cu solute atoms. The next work considers several possible configurations to calculate the interaction between point-defect pairs. Figure 4a shows the specific model used in this work.

### 2.2. Computational Details

This section of the first-principles calculations of this work is based on density functional theory, implemented in the Vienna ab-initio simulation package (VASP) [31,32]. The interaction of ions and electrons was described by projector augment wave (PAW) [33,34] pseudo-potentials. The exchange-correlation function was constructed by the generalized gradient approximation (GGA) with the Perdew–Burke–Ernzerhof (PBE) [35]. All structures were fully relaxed with respect to atomic positions as well as all lattice parameters in order to find the lowest-energy structure. The cutoff energy of the electron-wave function in plane waves is 450 eV. Atoms relax until their residual force converges to 0.01 eV/Å. The total energy was converged to 1.0 × 10^−5^ eV/atom during the optimization. All the unit cell structure models for the GP zone were constructed using the Monkhorst–Pack method [36] to generate a *k*-point grid, with atoms assigned to about 7000 wave vector points at each reciprocal grid point [37]. For the interaction of point defects with the GP zone, the first Brillouin zone was sampled using 7 × 13 × 1 Monkhorst–Pack *k*-point grids. In addition, a 3 × 3 × 8 *k*-point grid and a 2 × 1 × 11 supercell were used to calculate the “interaction energy” (detailed definitions are explained below).

The supercell containing 88 atoms was used to describe the interaction of the point defects (Cu solute atoms and vacancies) with the GP zones ({MgSi} (100) layer in the Al matrix) in Al-Mg-Si(-Cu) alloy. The supercell consists of 60 Al atoms and a 2 × 1 × 3 {MgSi} GP zones, as shown in Figure 4b. The energy difference is defined as follows:

(1) $$\Delta E\left(X,i\right)=E_{tot}^{X,i}-E_{tot}^{X,B}$$

where $E_{tot}^{X,B}$ is the supercell that has Cu atoms or vacancies at the *B* position in the Al matrix away from the GP zones, and $E_{tot}^{X,i}$ is the supercell with the same point defect near or inside the GP zones at *i* = {1, 2, …} positions.

The formula for studying the interaction between vacancies within the GP zones and point defects is similar to (1). The interaction energy is defined as follows:

(2) $$\Delta E_{int}\left(X,i\right)={\left[E_{tot}^{X,i}-E_{tot}^{X,B}\right]}_{Vac}$$

where the subscript *Vac* indicates that there is a vacancy in the GP zones, $E_{tot}^{X,B}$ is the supercell with a Cu atom at position B, and $E_{tot}^{X,i}$ is the supercell with a Cu solute atom at *i* = {1, 2, 3, 4} positions.

The four-parameter Birch–Murnaghan equation of state and its linear form [38] were used to estimate the equilibrium total energy ($E_{0}$) and equilibrium volume ($V_{0}$).

(3) $$E\left(V\right)=a+bV^{-2/3}+cV^{-4/3}+dV^{-2}$$

where *a*, *b*, *c*, and *d* are fitting parameters. More details can be found in our previous work [39].

The formation enthalpy can be defined with respect to the solid solution [40]. Initially, Mg and Si atoms exist at substitution sites in the face-centered cubic (fcc) lattice of Al. Considering the fcc arrangement in solid solution, the formation enthalpy in this work is defined in terms of fcc Mg and fcc Si (rather than hcp Mg and diamond Si). Since Mg and Si do not have stable fcc structures, their formation enthalpies, relative to the solid solution (*SS*), are calculated as follows:

(4) $$\begin{array}{c} \Delta H_{ss}^{\mathrm{form}}\left({\mathrm{Mg}}_{a}{\mathrm{Al}}_{b}{\mathrm{Cu}}_{c}{\mathrm{Si}}_{d}\right)=E\left({\mathrm{Mg}}_{a}{\mathrm{Al}}_{b}{\mathrm{Cu}}_{c}{\mathrm{Si}}_{d}\right)-aE^{\mathrm{sub}}\left(\mathrm{Mg}\right)\\ -bE\left(\mathrm{Al}\right)-cE\left(\mathrm{Cu}\right)-dE^{\mathrm{sub}}\left(\mathrm{Si}\right) \end{array}$$

where $E^{\mathrm{sub}}\left(\mathrm{Mg}\right)$ and $E^{\mathrm{sub}}\left(\mathrm{Si}\right)$ are the enthalpy of substituting Al atoms by Mg and Si atoms, respectively. In a 3 × 3 × 3 Al supercell consisting of 1 Mg or Si atom and 107 Al atoms with a *k*-point grid of 5 × 5 × 5, $E^{\mathrm{sub}}\left(\mathrm{Mg}\right)$ and $E^{\mathrm{sub}}\left(\mathrm{Si}\right)$ were calculated. The substitution enthalpy of Mg atom is defined as:

(5) $$E^{\mathrm{sub}}\left(\mathrm{Mg}\right)=E\left({\mathrm{Al}}_{107}\mathrm{Mg}\right)-107/108E\left(\mathrm{Al}\right)$$

where $E\left(\mathrm{Al}\right)$ is the calculated total energy of the 3 × 3 × 3 Al supercell. The definition of $E^{\mathrm{sub}}\left(\mathrm{Mg}\right)$ was also feasible for $E^{\mathrm{sub}}\left(\mathrm{Si}\right)$.

In order to compare configurations with different Al contents, the formation enthalpy can also be expressed in kJ/mol of solute atoms. This transformation is achieved as follows: $\Delta H_{SS}\left[\mathrm{kJ}/\mathrm{mol}\;\mathrm{solute}\right]=\Delta H_{SS}\left[\mathrm{kJ}/\mathrm{mol}\right]/\left(x\mathrm{Mg}+x\mathrm{Si}+x\mathrm{Cu}\right)$, where xMg, xSi, and xCu are the atomic fractions of Mg, Si, and Cu in the atomic configuration ${\mathrm{Mg}}_{a}{\mathrm{Al}}_{b}{\mathrm{Cu}}_{c}{\mathrm{Si}}_{d}$. This is the common definition of the formation enthalpy in the literature [40,41,42,43].

## 3. Results and Discussion

### 3.1. Solute Atom Concentration in GP Zones

**Figure 5:**
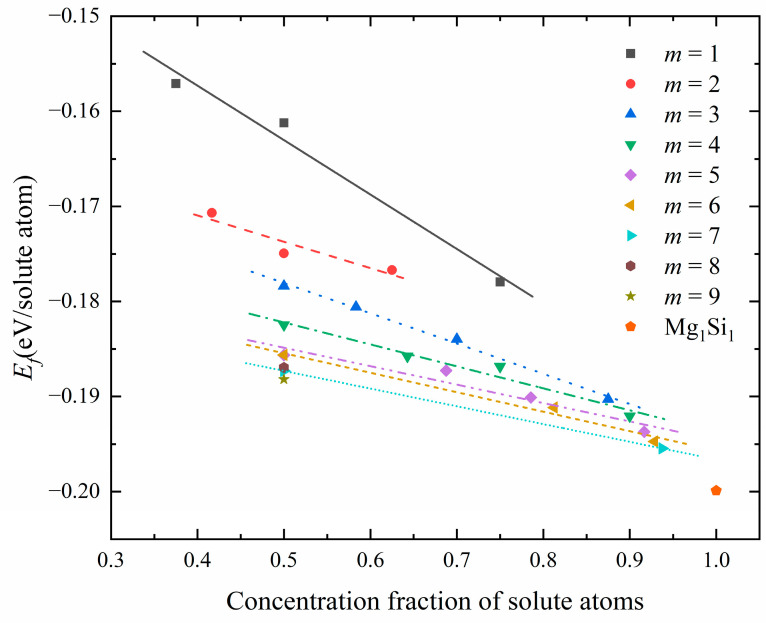
The relationship between solute atom concentration and formation enthalpy in the atomic configuration of GP zones corresponding to different Mg/Si/Mg unit cell numbers *m*.

Table 1 displays the atomic configurations, solute atomic concentration, and formation-enthalpy information of interest. The relationship between the solute atomic concentration (i.e., the total concentration of Mg and Si atoms) and the formation enthalpy is visualized in Figure 5. The scattered points in the figure correspond to the calculated formation enthalpy of a specific configuration at a given solute atom concentration, and the straight lines are obtained by linear fitting of the same Mg/Si/Mg unit cell number *m*.

Figure 5 shows the calculated formation enthalpies for all GP-zone configurations. The Mg_1_Si_1_ atomic configuration, with a solute atomic concentration of 100%, exhibits the lowest formation enthalpy. For configurations with the same *m* of Mg/Si/Mg cell, the formation enthalpy in the GP zones decreases as the solute atom concentration increases. From the perspective of thermodynamics, structures lacking Al atoms in the {MgSi} (100) layer are more stable. Moreover, it is observed that as *m* increases, the straight line exhibits a greater negative slope along the coordinate axis, indicating that the formation enthalpy decreases with an increase in the (100) plane cross-section area of GP zones, for a given solute atom concentration.

**Figure 6:**
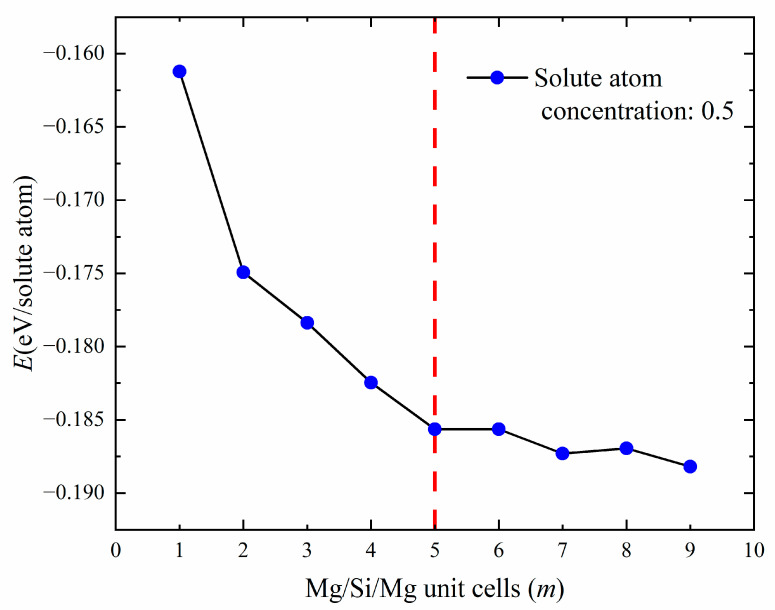
The line graph of the relationship between the number of Mg/Si/Mg unit cells *m* and the formation enthalpy (eV/solute atom) when the solute atom concentration is 0.5.

Next, this study investigated whether there is an optimal or convergent value in terms of thermodynamics for increasing the (100) plane cross-sectional area of GP zones. The diffusivity of Mg and Si atoms in the solid solution was considered to influence the formation of precipitated-phase structures. The earliest precipitated phase contains a large number of Al atoms [17,44]. To obtain size information for GP zones, an additional constraint was applied, selecting only structures with a 50% solute atomic concentration (Mg + Si) for calculation [28]. Figure 6 shows the relationship between the cross-sectional area of GP zones and the formation enthalpy. It was found that the formation enthalpy essentially converges at *m* = 5, indicating that the GP zone’s size tends to stay at about 2 nm (*m* = 5, 19.972 Å) during the aging precipitation process. It is consistent with previous experiments reporting [18], indirectly proves the {MgSi} substructure atomic model in this work is reasonable.

### 3.2. Lattice Mismatch in GP Zones

**Figure 7:**
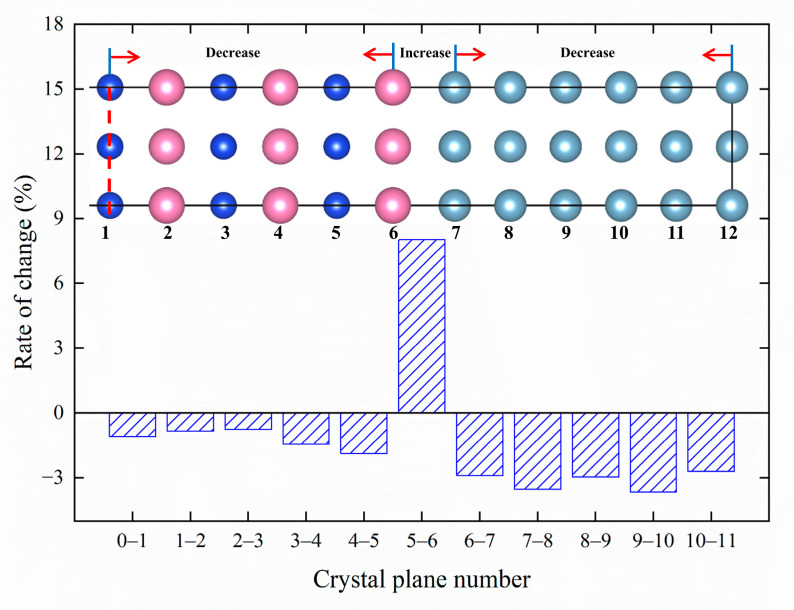
The change rate of interplanar spacing along the (001) direction of GP zones in Al-Mg-Si alloys.

The presence of {MgSi} (100) in the Al matrix results in a slight distortion of the fcc lattice. In order to understand the formation and transformation processes of GP zones, it is necessary to quantify their behavior. In this work, we investigate the atomic-layer displacement of Mg_6_Si_5_Al_11_ after relaxation for *m* = 5 atomic configuration (GP zone size approximately 2 nm). The crystal structure information before and after the relaxation of the Mg_6_Si_5_Al_11_ configuration is shown in Table 2. The crystal structure information before relaxation is based on the Al matrix crystal structure. Compared to before relaxation, the formation of GP zones causes an increase of 3.58% in the Mg_6_Si_5_Al_11_ unit cell volume, and an increase of 2.42% in the lattice constants *a* and *b* while *c* decreases by 1.25%. The alternating Mg and Si (001) planes likely cause the contraction of *c* and the expansion of *a* and *b*, resulting in a relatively stable unit cell volume. The structure information of the {MgSi} layer is also listed in Table 3 and similar changes in the lattice constants *a* and *b*, and a decrease of 1.20% in *c,* are observed. These predicted lattice parameters are consistent with the results obtained by Liu et al. using geographic phase analysis (GPA) [20]. In order to refine the changes in (001) interplanar spacing and atomic displacement after GP zone formation, the atomic-layer displacement statistics are summarized in Table 4, and the change rate of (001) interplanar spacing is visualized in Figure 7, with the atomic-layer numbers marked.

Figure 7 shows the atomic model of Mg_6_Si_5_Al_11_, and the labels 0-11 are the atomic layer numbers. It should be noted that due to the symmetry of the crystal structure, only half of the atomic model with zero atomic layer as the symmetry plane is shown in the figure. Figure 7 indicates that the interlayer distances between Mg and Si (001) crystal planes decrease, while the interlayer distances between nearby Al (001) crystal planes also decrease and the distance between Mg atomic layer and Al atomic layer increases. Furthermore, the lattice constants *a*_Mg_ = 4.515 Å for fcc Mg and *a*_Al_ = 4.043 Å for fcc Al are calculated in this work. The interlayer spacing between the fifth and sixth layers is 2.184 Å, where *a*_Mg_/2 = 2.2575 Å, *a*_Al_/2 = 2.0215 Å. *a*_Mg_/2 < 2.184 Å < *a*_Al_/2. It indicated that the Mg atomic layer and Al atomic layer interlayer distances are between the fcc Mg (001) interplanar spacing and the fcc Al (001) interplanar spacing, which is consistent with Vegard’s Law [45].

### 3.3. Growth Mechanism of GP Zones

**Figure 8:**
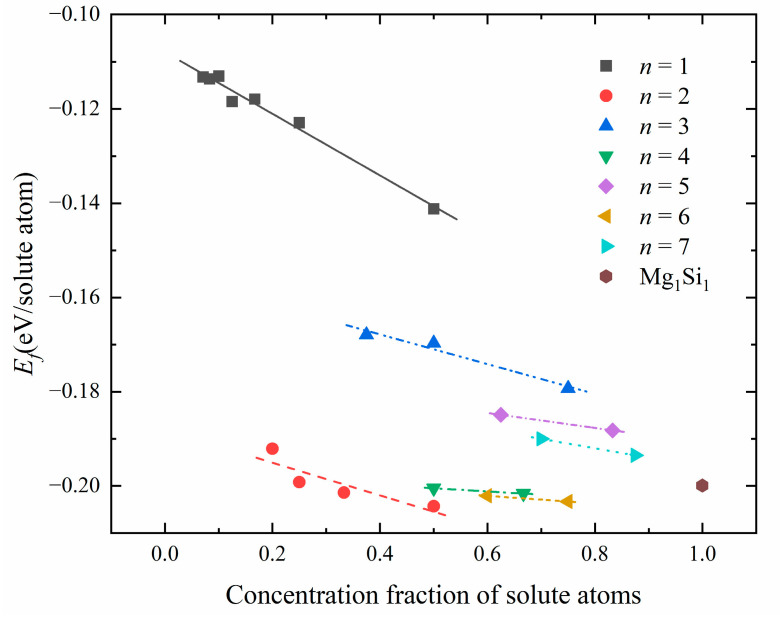
The relationship between solute atom concentration and formation enthalpy in atomic configurations corresponding to different number *n* of {MgSi} layers.

Table 5 shows the number *n* of {MgSi} layers, solute atom concentration, and formation enthalpy for different atomic configurations in GP zones. Figure 8 shows the relationship between solute atom concentration and formation enthalpy in atomic configurations corresponding to a different number of {MgSi} layers. As depicted in Figure 8, for configurations with the same {MgSi} layer number *n*, the formation enthalpy of GP zones decreases with the increase of the solute atom concentration. It is indicated that the aggregation of solute atoms and the growth of GP zones occur during the early stage of the aging precipitation process. When studying the relationship between the number of {MgSi} layers *n* and the formation enthalpy, it can be found that the formation enthalpies of {MgSi} in the odd layers (*n* = 1, 3, 5, 7) are higher than Mg_1_Si_1_ atomic configuration. However, the formation enthalpies of {MgSi} in the even layers (*n* = 2, 4, 6) are lower than or close to the Mg_1_Si_1_ atomic configuration. The results show that in the GP zones, the stability of the {MgSi} layers with even layers is higher than the odd layers. Since the structure stability of the {MgSi} layers with even layer numbers is slightly greater than that of the Mg_1_Si_1_ atomic configuration without Al atoms, some Al contents are present along the GP-zones (100) direction, and the Al atomic layer can alleviate the strain caused by the {MgSi} atomic layer.

**Figure 9:**
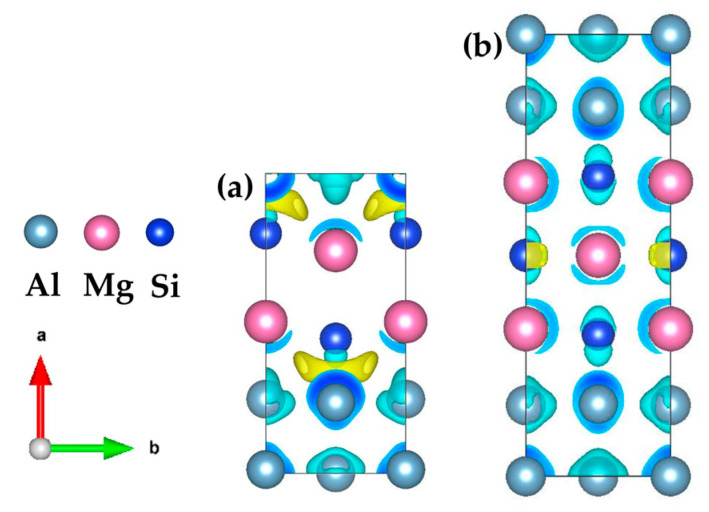
Three-dimensional charge-density difference map of (**a**) {MgSi}_2_Al_4_ and (**b**) {MgSi}_3_Al_6_, with an isosurface value of 0.0055 e/Å^3^. (The variables a, b represent the *x*, *y* axes, respectively, while the arrows indicate the positive directions.)

In order to study the mechanism that the even {MgSi} (100) atomic layer is more stable than the odd {MgSi} (100) atomic layer in the GP zones with the same solute atom concentration. The electronic structure of the two-layer {MgSi}_2_Al_4_ and three-layer {MgSi}_3_Al_6_ are analyzed. Figure 9 shows the three-dimensional charge-density difference map of the two configurations of GP zones. The yellow areas represent the gain of charge and the blue areas represent the loss of charge. It can be clearly seen from the figure that there is obvious charge aggregation between the {MgSi} (100) atomic layer and the Al atomic layer in the {MgSi}_2_Al_4_ configuration with even layers. It results in strong interaction between the {MgSi} (100) atomic layer and the Al atomic layer, especially between Si and Al atoms. The Al atom obviously loses its charge on the side close to the Si atom, and the charge is transferred to form strong interactions between Si atoms and Al atoms. At the same time, Si atoms are attracted to the Al atom layer, and the distance between Si and Al atoms decreases, which enhances bond cooperation.

Since the {MgSi} atomic layer has a strong bonding effect on the Al matrix, it is favorable for the {MgSi} atomic layer to attach and grow on the Al matrix. In the odd-layer {MgSi}_3_Al_6_ configuration, the charges are more delocalized, and only a small amount of localization phenomena exists near the Si atom, resulting in weaker bond cooperation than the even-layer {MgSi}_2_Al_4_ configuration. We speculate that the even-layer {MgSi} atomic layer is more stable than the odd-layer {MgSi} atomic layer in the GP zones, the stronger electronic interaction between the {MgSi} atomic layer and the Al atomic layer is an important reason.

**Figure 10:**
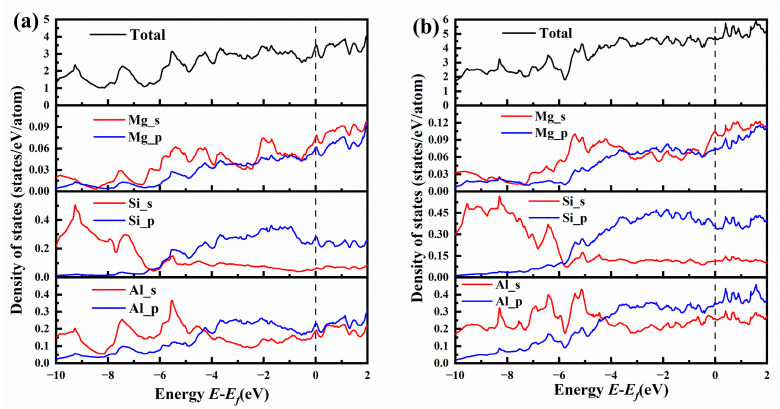
The total and partial electronic density of states (TDOSs and PDOSs) for (**a**) {MgSi}_2_Al_4_ and (**b**) {MgSi}_3_Al_6_.

Figure 10 shows the total and partial electronic density of states for {MgSi}_2_Al_4_ and {MgSi}_3_Al_6_. Comparing the total electronic density of states (TDOS) in Figure 10a,b, it can be found that the TDOS in Figure 10a has a more obvious peak than Figure 10b, indicating that the charge in the {MgSi}_2_Al_4_ configuration is more localized. Combined with the charge-density difference map, it can be found that the charges in the system are more concentrated, which allows the atoms near the charge to interact more strongly with each other.

Comparing the partial electronic density of states (PDOS) of Figure 10a,b, it can be found that the Mg-s state electrons in {MgSi}_2_Al_4_ have more obvious sharp peaks than those in {MgSi}_3_Al_6_, such as the Mg-s state and the Si-s state, Al-s state and Al-p state all form hybridization peaks at −7.5 eV, and Mg-s state forms hybridization peaks with Si-p state and Al-p state at −5 eV to 3 eV. Hybridization peaks are formed between Mg-s state and Al-s state at −2 eV, Mg-s/p state with Si-p state and Al-s/p state form hybrid peaks at Fermi level. More hybridization peaks mean stronger interatomic interactions, which further validates the conclusion from the formation-enthalpy calculation results that the even-layer {MgSi} has higher stability than the odd-layer {MgSi}.

**Figure 11:**
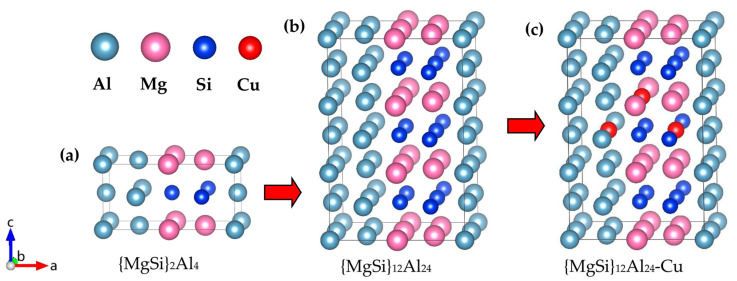
The most energetically favorable {MgSi}_2_Al_4_ configuration in the calculated GP-zones configuration and the atom model of Cu atom replacing other atoms. (**a**) The {MgSi}_2_Al_4_ configuration, (**b**) the 2 × 1 × 3 supercell of {MgSi}_2_Al_4_, and (**c**) the atom model in which one Cu atom replaces Al, Mg, and Si atoms, respectively. (The variables a, b, and c represent the *x*, *y*, and *z* axes, respectively, while the arrows indicate the positive directions.)

The above results proved that {MgSi}_2_Al_4_ was the most stable GP-zones structure among the configurations calculated in this work, as shown in Figure 11a. In order to study the substitution order of Cu atoms for other atoms in the GP zones, a 2 × 1 × 3 supercell of the {MgSi}_2_Al_4_ configuration was constructed, as shown in Figure 11b. Cu atoms are placed at the P_Al_, P_Mg_, and P_Cu_, as shown in Figure 11c. The calculated results of the formation enthalpy (eV/solute atom) under the three conditions are shown in Table 6, and the formation enthalpy of {MgSi}_2_Al_4_ is also given in the Table 6. The calculation results show that the formation enthalpy of the Mg_6_Si_6_Al_11_Cu configuration after the Cu atom replaces the Al atom decreases from the original −0.2043 eV/solute atom to −0.2054 eV/solute atom. However, when Cu atoms enter GP zones, the overall energy will increase correspondingly. The substitutional atom preference of the Cu atom in GP zones is Al > Si > Mg, indicating that the precipitation phase is accompanied by Cu atoms replacing Al atoms during the aging precipitation process, and the structure of the {MgSi} atomic layer is not easily influenced by the Cu atom.

### 3.4. Point Defects Interact with GP Zones

**Figure 12:**
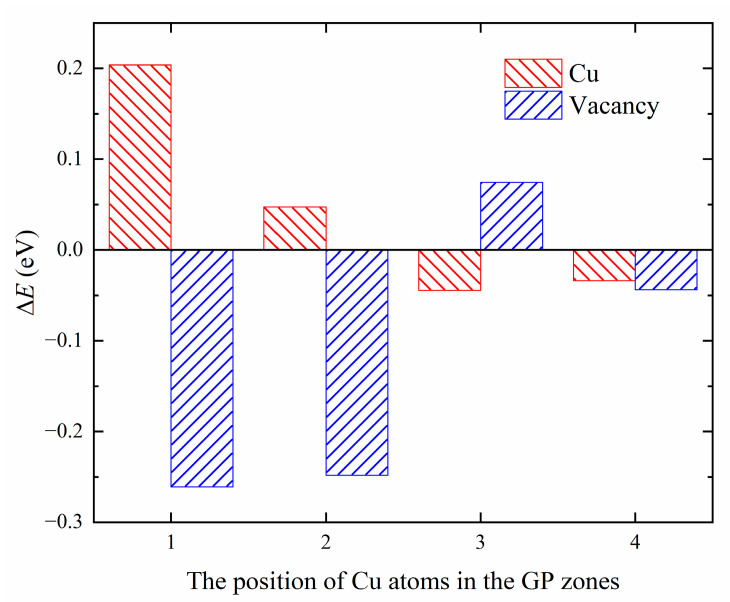
The energy difference of Cu/Vac defects at different positions.

We have computed the energy difference ($\Delta E\left(X,i\right)$) between two configurations, one in which a point defect, *X*, is located near of the GP zones, and the other in which the point defect is located in the bulk of aluminum, as shown in Figure 12. The positive value of $\Delta E\left(X,i\right)$ indicates that the point defect exhibits a repulsive effect with the GP zones at position *i*, while a negative value indicates attractive effects. A smaller value of $\Delta E\left(X,i\right)$ indicates a greater tendency of point defect *X* to occupy position *i*.

The results presented in Figure 12 indicate that the Cu solute atoms tend to be located in the Al matrix near the GP zones, showing weak attraction (−0.0445 eV). Vacancies tend to be captured by the GP zones, with a preference for replacing Si atomic sites (−0.2611 eV) to form quenched vacancies. Mg atomic sites serve as a second choice for vacancies (−0.2482 eV). Previous studies by Murayama et al. [22] suggest that in the spherical GP zones, Mg:Si ≈ 1, and Cu atoms are not enriched in the GP zones. The chemical properties of GP zones in the quaternary Al-Mg-Si-Cu alloy are consistent with those in the ternary Al-Mg-Si alloy, with the addition of Cu not altering the chemical properties of the GP zones. Additionally, Matsuda et al. [46] employed transmission electron microscope (TEM) studies to show that Cu atoms tend to segregate at the interface between the precipitation phase and the matrix. The phenomenon of lattice mismatch-induced segregation of copper (Cu) atoms has been investigated by various studies. Man et al. [47] conducted a 3D atom-probe (3DAP) analysis, which confirmed that Cu atoms are attracted to the lattice mismatch region. Weng et al. [48] observed the segregation of Cu atoms at the interface between the precipitate phase and α (Al) matrix using high-angle annular dark field-scanning transmission electron microscopy (HAADF-STEM). Among the four elements Al, Mg, Si, and Cu, the atomic radius of Cu is the smallest, leading to the release of coherent strain between interfaces, which is consistent with the results of our calculations indicating that Cu atoms tend to distribute in the Al matrix.

Regarding the interaction between Cu solute atoms in Al-Mg-Si-Cu and vacancies within the GP zones, as shown in Table 7, it can be observed that the absolute value of the interaction energy between Cu atoms and GP zones decreases when the GP zones contain Cu atoms. It is indicated that the interaction between Cu atoms and GP zones decreases due to the presence of vacancies, thereby reducing the effect of attraction and repulsion at each of the same locations. However, Cu atoms still exhibit a tendency to aggregate in the Al atom layer near the GP zones, and the presence of vacancies does not significantly alter the position preference of Cu atoms.

## 4. Conclusions

This paper explores the structure of GP zones and the interaction between point defects and GP zones in Al-Mg-Si(-Cu) alloys, based on considerations of structural stability. The paper theoretically predicts the atomic-structure models of GP zones and their growth mechanism in Al-Mg-Si alloys. Finally, the paper discusses the occupancy of Cu atoms and vacancy defects in the early stages of aging. The following conclusions have been drawn:

(1) This study has determined that the solute atom content of the {MgSi} (100) layer in the GP zones is 100%, which means that the structure without Al atoms in the {MgSi} (100) layer is the most stable. In the (100) direction of GP zones, the GP zones tend to grow in even layers and contain a certain Al content to relieve strain. Among all configurations in this study, {MgSi}_2_Al_4_ is the most stable GP-zone configuration, which is composed of two {MgSi} layers. Van Huis et al. [28] demonstrated in their research that the structures of {MgSi}_2_Al_10_, {MgSi}_1_Al_10_, Mg_3_Si_2_Al_5_, and Mg_2_Si_1_Al_3_; this means that there are Al atoms in the (100) direction of GP zones. This provides further support for our conclusions.
(2) The relaxation results of GP zones indicate that, compared to the Al matrix, the lattice parameters *a* and *b* of the GP zones increased by 2.42% and *c* decreased by 1.25%. Additionally, the study revealed that during the aging precipitation process, the size of GP zones tends to grow up to 2 nm. These predicted lattice parameters are consistent with the lattice parameter evolution trends revealed by GPA [18], which also provide validation for the rationale of our current investigation.
(3) The occupancy tendencies of point defects in GP zones exhibit distinct characteristics. Cu atoms demonstrate a tendency to segregate in the Al matrix surrounding GP zones, while vacancies have a tendency to be captured by the GP zones. It is observed that the tendencies of Cu atoms to occupy positions near GP zones remain unaltered during the early stage of aging, even when vacancies are trapped within the GP zones.

## Figures and Tables

**Table 1:** The number *m* of the Mg/Si/Mg unit cell, solute atom concentration, and formation enthalpy corresponding to different atomic configurations in GP zones.

Configuration	Mg/Si/Mg Unit Cell Number *m*	*x*_solute_	*E* (eV/Solute Atom)
Mg_1_Si_1_	—	1.000	−0.1999
Mg_2_Si_1_Al_1_	1	0.750	−0.1779
Mg_2_Si_1_Al_3_	1	0.500	−0.1612
Mg_2_Si_1_Al_5_	1	0.375	−0.1571
Mg_3_Si_2_Al_3_	2	0.625	−0.1767
Mg_3_Si_2_Al_5_	2	0.500	−0.1749
Mg_3_Si_2_Al_7_	2	0.417	−0.1707
Mg_4_Si_3_Al_1_	3	0.875	−0.1903
Mg_4_Si_3_Al_3_	3	0.700	−0.1840
Mg_4_Si_3_Al_5_	3	0.583	−0.1806
Mg_4_Si_3_Al_7_	3	0.500	−0.1784
Mg_5_Si_4_Al_1_	4	0.900	−0.1921
Mg_5_Si_4_Al_3_	4	0.750	−0.1868
Mg_5_Si_4_Al_5_	4	0.649	−0.1858
Mg_5_Si_4_Al_9_	4	0.500	−0.1825
Mg_6_Si_5_Al_1_	5	0.917	−0.1937
Mg_6_Si_5_Al_3_	5	0.786	−0.1901
Mg_6_Si_5_Al_5_	5	0.688	−0.1873
Mg_6_Si_5_Al_11_	5	0.500	−0.1856
Mg_7_Si_6_Al_1_	6	0.929	−0.1947
Mg_7_Si_6_Al_3_	6	0.813	−0.1912
Mg_7_Si_6_Al_13_	6	0.500	−0.1856
Mg_8_Si_7_Al_1_	7	0.938	−0.1955
Mg_8_Si_7_Al_15_	7	0.500	−0.1873
Mg_9_Si_8_Al_17_	8	0.500	−0.1870
Mg_10_Si_9_Al_19_	9	0.500	−0.1882

**Table 2:** Changes of lattice constants before and after Mg_6_Si_5_Al_11_ atomic configuration relaxed in Al-Mg-Si alloys.

Mg_6_Si_5_Al_11_	*a* (Å)	*b* (Å)	*c* (Å)	*V* (Å^3^)
Initial	4.043	4.043	44.473	726.949
Relaxed	4.141	4.141	43.917	752.998
Rate of change	2.42%	2.42%	−1.25%	3.58%

**Table 3:** Changes of lattice constants before and after GP zones relaxed in Al-Mg-Si alloys.

{MgSi} Atomic Layer	*a* (Å)	*b* (Å)	*c* (Å)	*V* (Å^3^)
Initial	4.043	4.043	20.215	330.431
Relaxed	4.141	4.141	19.972	342.477
Rate of change	2.42%	2.42%	−1.20%	3.65%

**Table 4:** Lattice mismatch information along the (001) direction in the GP zones of Al-Mg-Si alloys.

The Number of (001) Plane	Variation of (001) Interplanar Spacing	Change Rate of (001) Interplanar Spacing	Atomic-Layer Displacement
1	−0.02176	−1.08%	−1.08%
2	−0.01693	−0.84%	−0.96%
3	−0.01561	−0.77%	−0.90%
4	−0.02923	−1.45%	−1.03%
5	−0.03801	−1.88%	−1.20%
6	0.16225	8.03%	0.34%
7	−0.05865	−2.90%	−0.13%
8	−0.07139	−3.53%	−0.55%
9	−0.05996	−2.97%	−0.82%
10	−0.07402	−3.66%	−1.10%
11	−0.05469	−2.71%	−1.25%

**Table 5:** The number *n* of {MgSi} layers, solute atom concentration, and formation enthalpy corresponding to different atomic configurations in GP zones.

Configuration	Number of Layers (*n*)	x_solute_	*E* (eV/Solute Atom)
{MgSi}Al_2_	1	0.500	−0.1412
{MgSi}Al_6_	1	0.250	−0.1229
{MgSi}Al_10_	1	0.167	−0.1179
{MgSi}Al_14_	1	0.125	−0.1184
{MgSi}Al_18_	1	0.100	−0.1130
{MgSi}Al_22_	1	0.083	−0.1136
{MgSi}Al_26_	1	0.071	−0.1132
{MgSi}_2_Al_4_	2	0.500	−0.2043
{MgSi}_2_Al_8_	2	0.333	−0.2014
{MgSi}_2_Al_12_	2	0.250	−0.1992
{MgSi}_2_Al_16_	2	0.200	−0.1921
{MgSi}_3_Al_2_	3	0.750	−0.1793
{MgSi}_3_Al_6_	3	0.500	−0.1697
{MgSi}_3_Al_10_	3	0.375	−0.1679
{MgSi}_4_Al_4_	4	0.667	−0.2016
{MgSi}_4_Al_8_	4	0.500	−0.2005
{MgSi}_5_Al_2_	5	0.833	−0.1882
{MgSi}_5_Al_6_	5	0.625	−0.1849
{MgSi}_6_Al_4_	6	0.750	−0.2033
{MgSi}_6_Al_8_	6	0.600	−0.2021
{MgSi}_7_Al_2_	7	0.875	−0.1935
{MgSi}_7_Al_6_	7	0.700	−0.1900

**Table 6:** The formation enthalpy of the configurations corresponding to the substitution of different atoms by Cu atoms in the GP zones in Al-Mg-Si(-Cu) alloys.

Configurations	Substitution Atoms	*E* (eV/Solute Atom)
{MgSi}_2_Al_4_	—	−0.2043
Mg_6_Si_6_Al_11_Cu	Al	−0.2054
Mg_5_Si_6_Al_12_Cu	Mg	−0.1882
Mg6Si_5_Al_12_Cu	Si	−0.1946

**Table 7:** The interaction energy of Cu defects at different positions.

Position *i*	Cu-Vac (eV/Solute Atom)
1—inside Si layer	0.2500
2—inside Mg layer	0.0023
3—next-nearest layer	−0.0404
4—next-nearest layer	−0.0236

## Data Availability

Data are contained within the article.
